# Exposure to Endocrine Disrupting Chemicals and Male Reproductive Health

**DOI:** 10.3389/fpubh.2014.00055

**Published:** 2014-06-05

**Authors:** Hueiwang Anna Jeng

**Affiliations:** ^1^School of Community and Environmental Health, College of Health Sciences, Old Dominion University, Norfolk, VA, USA

**Keywords:** endocrine disruptors, bisphenol A, phthalates, polychlorinated biphenyls, dichlorodiphenyltrichloroethane, dichlorodiphenyldichloroethylene

## Abstract

Endocrine disrupting chemicals (EDCs) can interfere with normal hormonal balance and may exert adverse consequences on humans. The male reproductive system may be susceptible to the effects of such environmental toxicants. This review discusses the recent progress in scientific data mainly from epidemiology studies on the associations between EDCs and male reproductive health and our understanding of possible mechanisms associated with the effects of EDCs on male reproductive health. Finally, the review provides recommendations on future research to enhance our understanding of EDCs and male reproductive health. The review highlights the need for (1) well-defined longitudinal epidemiology studies, with appropriately designed exposure assessment to determine potential causal relationships; (2) chemical and biochemical approaches aimed at a better understanding of the mechanism of action of xenoestrogens with regard to low-dose effects, and assessment of identify genetic susceptibility factors associated with the risk of adverse effects following exposure to EDCs.

## Introduction

Endocrine disrupting chemicals (EDCs) are estrogen-like and/or anti-androgenic compounds that disrupt and interfere with the production, release, transport, metabolism, binding, or elimination of natural hormones in the body responsible for the maintenance of homeostasis and the regulation of developmental processes (1). EDCs include persistent pesticides and herbicides, methoxychlor, biocides, heat stabilizers, and chemical catalysts, plastic contaminants, pharmaceuticals, or dietary components. Those exogenous compounds can arise from industrial and domestic effluents or agricultural and urban runoff. The general population continues to be exposed to EDCs through ingestion of contaminated food, inhalation of contaminated air and dust, and skin contact, while some areas are subjected to greater risk due to geographical and cultural reasons (2). Due to temporal downward trends in semen quality and testosterone levels and increased rates of testicular cancers among adult male populations (3, 4), scientific researchers, and the general public have become increasingly concerned regarding the potential risk of EDCs to men’s reproductive health. Cellular models and animal toxicological studies have demonstrated that EDCs can exert adverse effects on the male reproductive system. In humans, there are a growing number of epidemiological studies about EDCs and detrimental reproductive function. However, a potential decline in human male reproductive health and a link to exposure to endocrine active chemicals in the environment has been controversial for almost two decades. This article focuses on the review of the human data regarding the relationship between exposures to known or suspected EDCs. It specifically focuses on bisphenol A (BPA), phthalates, polychlorinated biphenyls (PCBs), and dichlorodiphenyltrichloroethane (DDT)/dichlorodiphenyldichloroethylene (DDE) and men’s semen quality, sperm DNA damage, reproductive hormone levels, and reproductive diseases. This review provides: (1) an introduction to several common EDCs and their sources, (2) an overview of the state of the human evidence for adverse impacts of EDCs on male reproductive health, and (3) a description of possible mechanisms of action for EDCs involved with detrimental male reproductive functions based on *in vitro* and *in vivo* studies, and (4) recommendations for future research needed to enhance our understanding of the effect of EDCs on male reproductive health.

## Endocrine Disruptors

Polychlorinated biphenyls are a class of synthetic chlorinated compounds, which have been recognized as EDCs. PCBs were used in industrial and consumer products such as transformers and hydraulic fluids, and as an additive in paints, oils, and building materials. The use and production of PCBs have been banned since the 1970s, however, there has been no decrease or a slight decrease in the environment since the middle of 1990s (5). There is still considerable health risk from human exposure to PCBs from consumption of contained foods (6).

Dichlorodiphenyltrichloroethane and its main metabolite (*p,p*′-DDE) is a widespread, persistent environmental contaminant (7). Technical grade DDT is a mixture of *p*,*p*′-DDT (85%), *o*,*p*′-DDT (15%), and *o*,*o*′-DDT (trace amounts). Both *p*,*p*′-DDT and *o*,*p*′-DDT promote estrogenic activity (8). Reproductive abnormalities attributed to DDT/DDE exposure have been reported in a variety of wildlife animals (9, 10) and in laboratory rats (11). Epidemiological studies have recently addressed the link between exposure to DDT and male reproductive health (12–15).

Bisphenol A has been extensively used in production of polycarbonate plastic, epoxy resin, food packaging, and lacquers for food cans. Human beings are primarily exposed to BAP via dietary ingestion of leachings from the inner lining of cans and microware containers during heating of food materials and via beverages in polycarbonate bottles due to repeated usage or contact with any acid/alkaline (16). BPA is prevalent in our environment and measurable levels have been detected in the majority of individuals. Estrogenic activity of BPA was confirmed and is the basis for the recognition of the compound as a known endocrine disruptor.

Phthalates are ubiquitous industrial chemicals that are reported to adversely affect human reproductive outcomes. They are divided into two distinct groups based on molecular weight and with very different applications, toxicological properties, and classification: high molecular weight compounds (di-2-ethylhexyl phthalate with alkyl chain lengths from 8 to 13 carbons) and low molecular weight compounds [diethyl phthalate, dibutyl phthalate (DBP)]. Phthalates also include three di-(2-ethylhexyl) phthalate (DEHP) metabolites, mono-(2-ethylhexyl) phthalate (MEHP) and two oxidative metabolites, mono-(2-ethyl-5-hydroxyhexyl) phthalate (MEHHP), and mono-(2-ethyl-5-oxohexyl) phthalate (MEOHP). High molecular weight phthalates are primarily used as plasticizers in the manufacture of flexible vinyl plastic which, in turn, is used in consumer products, flooring and wall coverings, and medical devices (17). Low molecular weight phthalates are used in personal-care products as solvents and plasticizers for cellulose acetate. Exposure through ingestion, inhalation, and dermal contact are considered important routes of exposure for the general population. Upon exposure, phthalates are rapidly metabolized and excreted in urine and feces (17). Measurement of urinary concentrations of phthalate metabolites has been used as the most common biomonitoring approach for investigating human exposure to phthalates.

## Associations between EDCs and Semen Quality

The testicle seems to be an important target organ for PCBs, which can disrupt sperm production and development (18). PCBs had a consistent, significant inverse association with sperm motility, while its relationship with sperm concentration was less inconsistent (Table 1). An association with lower sperm concentrations likely occurred only at higher PCB concentrations. Men who had serum PCB levels of 240 ng/g lipid and partners diagnosed with an inability to conceive a pregnancy were associated with lower sperm concentrations (19). Bonde et al. selected 2,2′,4,4′,5,5′-hexachlorobiphenyl (CB-153) as a marker of PCB congeners and found that the non-coplanar PCB congeners only affected sperm motility, whereas the coplanar dioxin-like PCB congener CB-77 also reduced sperm counts (20).

**Table 1:** **Epidemiological studies on semen quality and endocrine disruptors including phthalates, BPA, PCB, and DDT/DDE**.

Compound	Study design	Sample size and subjects	Age	Concentration	Semen quality	First author (year)
**PHTHALATE**						
DEP	Cross-sectional	300 Healthy males	29	0.64–3.11 μg/mL	↓Concentration	Pant et al. (21)
DBP	Cross-sectional	300 Healthy males	29	0.18–1.65 μg/mL	↓Concentration, motility	Pant et al. (21)
DBP	Cross-sectional	300 Healthy males	28–29	13.47 μg/mL	↓Motility, viability	Pant et al. (22)
DEHP	Cross-sectional	300 Healthy males	28–29	5.73 μg/mL	↓Motility, viability	Pant et al. (22)
MEP	Cross-sectional	168 Male partners of sub-fertile couples	36	175.5 ng/mL	No relationship	Duty et al. (23)
MEP	Cross-sectional	463 Male partners of sub-fertile couples	36	180 ng/mL	No association	Hauser et al. (24)
MEP	Cross-sectional	45 Male partners of sub-fertile couples	35	121.9 ng/mL	↓Concentration	Wirth et al. (25)
MEP	Cross-sectional	269 Male from infertility clinic	32	153.6 μg/mL	No relation	Jurewicz et al. (26)
MBP	Cross-sectional	168 Male partners of sub-fertile couples	36	16.1 ng/mL	↓Concentration, motility, morphology	Duty et al. (23)
MBP	Cross-sectional	463 Male partners of sub-fertile couples	36	17.3 ng/mL	↓Concentration, motility	Hauser et al. (24)
MBP	Cross-sectional	45 Male partners of sub-fertile couples	35	26.9 ng/mL	No association	Wirth et al. (25)
MBP	Cross-sectional	269 Males from infertility clinic	32	108.5 μg/mL	No association	Jurewicz et al. (26)
MEHP	Cross-sectional	463 Male partners of sub-fertile couples	36	8.0 ng/mL	No association	Hauser et al. (24)
MEHP	Cross-sectional	168 Male partners of sub-fertile couples	36	7.6 ng/mL	No association	Duty et al. (23)
MEHP	Cross-sectional	45 Male partners of sub-fertile couples	35	11.5 ng/mL	No association	Wirth et al. (25)
MEHP	Cross-sectional	269 Males from infertility clinic	32	18.4 μg/mL	↓Motility	Jurewicz et al. (26)
MMP	Cross-sectional	168 Male partners of sub-fertile couples	36	7.5 ng/mL	↓Morphology	Duty et al. (23)
MMP	Cross-sectional	463 Male partners of sub-fertile couple	36	3.6 ng/mL	No association	Hauser et al. (24)
MMP	Cross-sectional	45 Male partners of sub-fertile couples	35	1.1 ng/mL	No association	Wirth et al. (25)
MCPP	Cross-sectional	45 Male partners of sub-fertile couples	35	2.5 ng/mL	↓Morphology	Wirth et al. (25)
BPA	Cross-sectional	190 Male from infertility clinic	36	1.3 ng/mL	↓Concentration, motility, morphology	Meeker et al. (27)
BPA	Cross-sectional	375 Male partners of pregnant women	32 (18–53)	1.5 μg/L	No association	Mendiola et al. (28, 29)
BPA	Cross-sectional	218 Males	N/A	1.6–5.9 μg/L	↓Sperm concentration, motility, viability, count	Li et al. (30)
BPA	Prospective cohort	142 Male partners of sub-fertile couples	34	1.55 ng/mL	↓Concentration, vitality, count	Knez et al. (31)
PCB	Pilot study	29 Male partners of infertile/sub-fertile couples	33	242 ng/g lipids	↓Motility	Hauser et al. (19)
PCB	Cross-sectional	303 Male partners of sub-fertile couple	35	43 ng/g lipids	↓Motility	Hauser et al. (32)
PCB	Cross-sectional	303 Male partners of sub-fertile couples	35	223 ng/g lipids	↓Motility	Hauser et al. (32)
*p*,*p*′DDT	Cross-sectional	311 Healthy males	23 (18–40)	90.23 μg/g lipid	↓Motility	Aneck-Hahn et al. (33)
*p*,*p*′-DDE	Cross-sectional	24 Healthy male	21 (16–28)	77.9 μg/g lipid	↓Volume, count	Ayotte et al. (34)
*p*,*p*′-DDE	Cross-sectional	116 Healthy males	27	45.0 μg/g lipid	↓Motility, morphology	de Jager et al. (13)
*p*,*p*′-DDE	Case–control	73 Healthy males	25	1.05 μg/g lipid	No relationship	Charlier and Foidart (35)
*p*,*p*′-DDE	Cross-sectional	195 Healthy males	24–65	240 ng/g lipid (80–887)	No relationship	Rignell-Hydbom et al. (12)
*p,p*′-DDE	Cross-sectional	212 Sub-fertile males	28–45	220 ng/g lipid (72.5–7776)	No relationship	Hauser et al. (15)
*p,p*′-DDE	Pilot study	29 Male partners of infertile/sub-fertile couples	33	354 ng/g lipid	↓Motility	Hauser et al. (19)

*DBP, Di-*n*-butyl phthalate; DEHP, Di(2-ethylhexyl) phthalate; MEP, monoethyl phthalate; MbzP, monobenzyl phthalate; MBP, mono-*n*-butyl phthalate; MEHP, mono-2-ethylhexyl phthalate; MMP, mono-methyl phthalate; MEOHP, mono-(2-ethyl-5-oxohexyl) phthalate; MiBP, mono-isobutyl phthalate; MEHHP, mono-(2-ethyl-5-hydroxyhexyl) phthalate; MCPP, mono-(3-carboxypropyl) phthalate, N/A, no data available*.

The available epidemiological evidence suggests that exposure to high DDT concentrations has links to semen quality (Table 1). The studies in malaria-endemic areas, where DDT was sprayed consistently, show an association between higher serum DDT concentration and decreased semen quality (13, 33). However, such associations were not found in studies conducted in populations where DDT/DDE concentrations were lower. DDT/DDE negatively impacted sperm motility, morphology, count, and semen volume in a South African population of 311 young males living in a malaria area in the Limpopo Province (33). Other studies also observed that DDT exposure adversely affected sperm quality, mainly through decreased motility (12–15). DDT can negatively affect sperm quality, especially when high concentration levels are considered.

Various animal models of BPA exposure have shown multiple effects on the male reproductive system, including inhibition of the development of seminiferous tubules and spermatogenesis, and impaired semen quality (43, 44). Increasing evidence from epidemiological studies has revealed the relationship between exposure to BPA and sperm quality, however, that relationship seemingly occurs at high concentrations of BPA exposure. BPA concentration at 1.55 ng/mL in male partners of sub-fertile couples may influence sperm count (31) based on a reverse relationship between BPA concentration and sperm count. A cross-sectional study performed on a population of 20 healthy men showed that serum BPA level positively correlated with quick forward progression of sperm and was inversely correlated with the percentage of normal sperm (45). In a recent study of 360 fertile men, Mendiola et al. demonstrated that no correlation existed between urinary BPA levels and semen quality, while BPA was inversely correlated with free androgen index concentrations (28). Meeker et al. recorded a similar finding showing a non-significant trend of increasing BPA levels related to lower sperm concentration, motility, morphology, and higher levels of DNA damage (27). A follow-up study on 190 male partners attending an infertility clinic showed that urinary BPA concentration were not associated with semen quality parameters, but they were positively associated with sperm DNA damage (38).

Toxicological studies have consistently shown that phthalate metabolites are reproductive and developmental toxicants. There is evidence that pubertal and adult exposure to DEHP results in testicular toxicity and impaired spermatogenesis (46, 47). In the past decade, an increasing number of epidemiological studies have investigated associations between phthalate metabolites and semen quality, but results have not been consistent. One of the first studies investigated 168 men from sub-fertile couples and concluded that specific phthalate metabolites correlated with lower sperm concentration and motility. There were dose–response relations between mono-butyl phthalate (MBP) and semen quality (motility and concentration), but limited evidence existed for such relations between other phthalate metabolites and poor sperm morphology and concentration (23). In a follow-up study with emphasis on sperm motion parameters, the authors reported that monobenzyl phthalate (MBzP), MBP, and MEHP have associations with velocity, while no relationship was found for mono-methyl phthalate (MMP) and any sperm motion parameter (48). A recent re-analysis of their data with 463 men again found significant dose–response associations between MBP concentration and low sperm concentration and low motility (24). A recent study of 344 men who had normal semen concentration of 20–300 × 10^6^/mL showed that urinary phthalate metabolites [5OH-MEHP, MEHP, mono-isobutyl phthalate (MiBP)] levels were significantly associated with a decrease in sperm motility (26). Weak associations between exposure to phthalate metabolites and lower sperm concentration, motility and morphology in adults have been reported by several studies (24, 25, 48, 49); at the same time, some have not proven this connection to phthalate metabolites (MBP or MBzP) (50). The aforementioned studies have many important differences, including the age range of the study populations, healthy status of the study populations, differences in the analytical methods used to measure phthalates, adjustment for covariance, and exposure concentrations.

## DNA Integrity

Sperm DNA integrity represents an essential requirement for the accurate transmission of genetic information. The origin of human sperm DNA damage involves certain mechanisms, including (1) alterations in chromatin modeling during the process of spermiogenesis, (2) apoptosis, and (3) oxidative stress (51). Sperm DNA damage has been characterized using the Comet assay (39), the sperm chromatin structure assay (SCSA) (52), and terminal deoxynucleotidyl transferase-driven dUTP nick end labeling (TUNEL) assay (53). Methods assessing sperm DNA integrity have implications and applications for being a better predictor of both *in vivo* and *in vitro* fertility than the WHO sperm parameters (54).

There is limited and contradictory epidemiological evidence on whether PCBs can affect human sperm DNA (39, 52, 55). PCBs positively associated with percentage of DNA fragmentation analyzed by the TUNEL assay and the neutral Comet assay (20). Hauser et al. observed no statistically significant consistent associations between the Comet assay parameters and any of the individual PCB congeners or sum of PCBs (39). Reasons for this inconsistency might be different methodologies used to detect sperm DNA damage, varying exposure ranges to and mixtures of persistent organic compounds, and different inclusion criteria for the studies.

Spanò et al. studied whether *p,p*′-DDE was associated with altered sperm chromatin integrity among European men. They found no significant associations between *p,p*′-DDE serum concentrations and sperm chromatin integrity analyzed by the SCSA (41). A following study conducted by Stronati et al. also observed no correlation between *p,p*′-DDE and sperm DNA fragmentation of 652 adult Inuits. A study of a population of Mexican men exposed to a mean concentration of 45,000 ng/g lipid from DDT sprayed in the environment show that impaired sperm chromatin condensation was observed in 46.6% of participants (13). Another study that was characterized by a mean *p,p*′-DDE level of 90,230 ng/g for DDT reported 54.7% of the studied young men had impaired sperm chromatin condensation. The percentage of damaged sperm chromatin structure measured by the flow cytometric method had a weak, but positive relationship with DDT concentrations. However, the percentage of DNA damage and sperm chromatin structure defects were not correlated (56). The finding was in agreement with other studies (40, 52). Some epidemiological studies did not find any significant association between DDT/DDE exposure and human sperm DNA (39, 52, 53). However, all of the preceding studies were characterized by a relatively low level of exposure to DDT, as detected by the plasma analysis of DDE, and where the main route of exposure to DDT was through diet. Those studies suggested that additional factors (e.g., genetic background, lifestyle habits, characterization of actual DDT mixture, and their xenohormonal activities) need to be investigated in the future for a better understanding of the effect of DDT exposure on sperm DNA integrity.

A few studies with cellular models indicated that BPA has the potential to induce point mutation, double stranded DNA breaks, and aneuploidy (57, 58; Table 2). Human studies of BPA exposure and sperm DNA damage have been very limited. Meeker et al. assessed the relationship between urinary BPA concentrations and sperm DNA damage in men recruited through a United States infertility clinic. BPA has been associated with increased single-strand breaks of sperm DNA damage among men. Since the studied population was recruited through an infertility clinic, there is a limitation on the ability to generalize the results to the general population (27, 59). Another follow-up study investigating relationships between urinary concentrations of parabens and BPA and male reproductive health reported that both parabens and BPA were both positively associated with sperm DNA damage. The study did not reach conclusions regarding causal relationships due to the cross-sectional design (38).

**Table 2:** **Epidemiological. studies on DNA integrity and endocrine disruptors including phthalates, BPA, PCB, and DDT/DDE**.

Compound	Study design	Sample size	Age	Concentration	DAN integrity	First author (year)
**PHTHALATE**						
MEP	Cross-sectional	68 Male partners of sub-fertile couples	36	186.8 ng/mL	↑Tail distributed moment	Duty et al. (36)
MEP	Cross-sectional	379 Males from infertility clinic	36	171 ng/mL	↑DNA damage	Hauser et al. (37)
MEP	Cross-sectional	269 Males from infertility clinic	32	153.6 μg/mL	No association	Jurewicz et al. (26)
MBP	Cross-sectional	168 Male partners of sub-fertile couples	36	18.2 ng/mL	No association	Duty et al. (36)
MBP	Cross-sectional	379 Infertility clinic	36	17.9 ng/mL	↑DNA damage	Hauser et al. (37)
MBP	Cross-sectional	269 Males from infertility clinic	32	108.5 μg/mL	↑DNA damage	Jurewicz et al. (26)
MEHP	Cross-sectional	168 Male partners of sub-fertile couples	36	7.1 ng/mL	No association	Duty et al. (36)
MEHP	Cross-sectional	379 Infertility clinic	36	7.6 ng/mL	↑DNA damage	Hauser et al. (37)
MEHP	Cross-sectional	269 Males from infertility clinic	32	18.4 μg/mL	No association	Jurewicz et al. (26)
MMP	Cross-sectional	168 Males partners of sub-fertile couples	36	6.1 ng/mL	No association	Duty et al. (36)
MMP	Cross-sectional	379 Infertility clinic	36	3.6 ng/mL	No relation	Hauser et al. (37)
BPA	Cross-sectional	190 Males from infertility clinic	36	1.3 ng/mL	↑DNA damage	Meeker et al. (27)
BPA	Cross-sectional	132 Sub-fertile	37	Below limit of detection (LOD)	↑DNA damage	Meeker et al. (38)
PCB	Cross-sectional	212 Sub-fertile	28–45	226 ng/g lipid	No association	Hauser et al. (39)
PCB	Cross-sectional	707 Males from general population	34	180 ng/g lipid	↑DNA fragmentation	Spanò et al. (40, 41)
DDT/DDE	Cross-sectional	707 Healthy males	34	560 ng/g lipid	No relationship	Spanò et al. (40, 41)
*p*,*p*′-DDT	Cross-sectional	195 Healthy males	47	240 ng/g lipid (80–887)	No relationship	Rignell-Hydbom et al. (12)
*p,p*′-DDE	Cross-sectional	212 Sub-fertile males	28–45	220 ng/g lipid (72.5–7776)	No relationship	Hauser et al. (15)
*p,p*′-DDE	Cross-sectional	680 Partners of pregnant women	34	750 ng/g lipid	↑DNA damage	Giwercman et al. (42)
*p,p*′-DDE	Cross-sectional	707 Males from general population	34	790 ng/g lipid	No association	Spanò et al. (40, 41)

Limited animal and epidemiologic data exist on the potential general population effects of phthalate exposure on sperm DNA integrity. In a US study, an association between increased sperm DNA damage and MEP was found, but there were no associations with the other phthalate monoesters (36; Table 2). A follow-up study with a larger sample of men and measurement of two oxidative metabolites of DEHP reported consistent evidence of the previous findings that MEP, a metabolite of diethyl phthalate, was associated with increased DNA damage and MEHP, a metabolite of DEHP, was associated with DNA damage after adjustment for the oxidative DEHP metabolites (37). When analyzing six urinary phthalate metabolites from 344 men with normal semen concentrations (20–300 mln/mL) or slight oligozoospermia (15–20 × 10^6^/mL), Jurewicz et al. reported that urinary MBP levels were significantly associated with an increase in sperm DNA damage (26). The same research group extended the preliminary study by including a large sample of men and measurements of more phthalate metabolites MEHP and MEOHP, two oxidative metabolites of DEHP. Sperm DNA damage was associated with MEHP after adjusting for DEHP oxidative metabolites (26). By contrast, a Swedish study did not find associations between any of the phthalate monoesters and sperm DNA damage measured with the SCSA (50). A recent study of 232 general population men from a Reproductive Center in Chongqing, China, showed no association between phthalate metabolites and sperm DNA damage using the alkaline single-cell gel electrophoresis assay (60).

## Reproductive System Track

Two congenital anomalies are included in the definition of the testicular dysgenesis syndrome (TDS): cryptorchidism and hypospadias. Cryptorchidism is the failure of one or both testicles to descend into the scrotum, which likely occurs by 6 months of age (61), thus study designs rely only on diagnosis in the delivery room are sub-optimal. Hypospadias, the condition in which the opening of the urethra is on the ventral side of the penis rather than that at the tip of the glans penis, can be diagnosed reliably at birth. Hypospadias may arise during the first trimester of *in utero* life and is classified as mild (first degree) to severe (third degree), depending on where the urethra opens on the penis. Eight studies have examined the relationship between cryptorchidism and/or hypospadias and DDT and/or metabolites of DDT. Table 3 summarizes results from limited published studies that have examined the association between PCBs and DDT and either cryptorchidism or hypospadias. Two case–control studies of PCBs and cryptorchidism reported no relationship to risk (62, 63), whereas a third offered measured support (64). One ecologic study of PCBs and hypospadias reported an inverse association (42). Because the sample sizes of prior studies may have been too small to detect statistically significant differences, a follow-up study was conducted among a large, well-described population in which the serum samples were collected at a time when PCB levels in the United States were higher. This study analyzed PCBs in the third-trimester serum samples from the mothers of 230 sons with cryptorchidism, 201 sons with hypospadias, and 593 sons with neither condition between 1959 and 1965, and did not strongly support the hypothesis that PCBs are associated with cryptorchidism or hypospadias. Because population serum PCB levels at the time of sample collection were considerably higher than levels at present, it is unlikely that current PCB exposure is related to the development of either anomaly (65).

**Table 3:** **Case–control studies on relationships between cryptorchidism and hypospadias and endocrine disruptors including PCB and DDT/DDE**.

Compound	Country	Outcome	Biospecimen	Cases	Controls	Results	First author (year)
PCB	Germany	Cryptorchidism	Maternal sera	18	30	Null	Hosie et al. (66)
PCB	Faroe Islands	Cryptorchidism	Umbilical cord	19	176	Null	Mol et al. (63)
PCB	France	Cryptorchidism	Cord blood	67	84	Null	Brucker-Davis et al. (64)
		Cryptorchidism	Colostrum	56	69	
PCB	US	Cryptorchidism	Maternal sera	230	593	Null	McGlynn et al. (65)
		Hypospadias	Maternal sera	201	593	
PCB	Italy	Hypospadias	Maternal sera	37	21	Null	Giordano et al. (67)
PCB	US	Hypospadias	Maternal sera	20	28	Null	Carmichael et al. (68)
*p,p*′-DDT *o,p*′-DDE	Germany	Cryptorchidism	Maternal sera	18	30	*p* > 0.05/*p* > 0.05	Hosie et al. (62)
*p,p*′-DDT *o,p*′-DDE	Denmark and Finland	Cryptorchidism	Breast milk	62	68	*p* = 0.47/*p* = 0.97	Damgaard et al. (69)
*p,p*′-DDT *o,p*′-DDE	Spain	Cryptorchidism	Placenta	48	114	*p* > 0.05/*p* > 0.05	Fernandez et al. (70)
		Hypospadias	
*p,p*′-DDE	Germany	Cryptorchidism	Maternal sera	18	30	*p* > 0.05	Hosie et al. (62)
*p,p*′-DDE	US	Cryptorchidism	Maternal sera	219	552	*p* > 0.05	Longnecker et al. (71)
		Hypospadias	Maternal sera	199	552	*p* > 0.05	
*p,p*′-DDE	US	Cryptorchidism	Maternal sera	75	283	*p* > 0.05	Bhatia et al. (8)
		Hypospadias	Maternal sera	66	283	*p* > 0.05	
*p,p*′-DDE	Denmark and Finland	Cryptorchidism	Breast milk	62	68	*p* = 0.26	Damgaard et al. (69)
*p,p*′-DDE	France	Cryptorchidism	Cord blood	67	84	*p* = 0.43	Brucker-Davis et al. (64)
		Cryptorchidism	Colostrum	56	69	*p* = 0.11	
*p,p*′-DDE	Italy	Hypospadias	Maternal sera	37	21	*p* > 0.05	Giordano et al. (67)
*p,p*′-DDE	US	Hypospadias	Maternal sera	20	28	*p* = 0.80	Carmichael et al. (68)

In a rat model, DBP could prompt changes in testes and male reproductive accessory glands, hypospadias, cryptorchidism, retention of nipples, and reduced anogenital distance (48, 72). Studies on laboratory animals have shown that exposure to 500–750 mg of DBP/kilogram during the critical period of male reproductive development results in remarkable phenotypic alterations in normal development (73). At birth, males presented with reduced anogenital distance. In adulthood, the phenotypes included cryptorchidism, epididymal agenesis, testicular atrophy with germ cell loss, hypospadias, and absent or smaller seminal vesicles and prostate (74). In humans, evidence demonstrating a negative action of phthalates on the reproductive tract is also accumulating. A relationship between anogenital distance and maternal urinary concentrations of phthalate metabolites was noted in 85 boys studied by Swan et al. (3). That study investigated the effect of prenatal environmental exposure to phthalates on genital development in newborns.

## Cancers

Studies investigating an association of DDT with endometrial cancer (75) and prostate and testicular cancer (76) have been inconclusive or do not support an association. Recently, increased exposure to *p,p*′-DDE has been reported to associate with risk of both seminomatous and non-seminomatous testicular germ cell tumors (TGCTs) (77). This case–control study investigated pre-diagnostic serum samples from 754 case subjects and 928 control subjects enrolled in the US Servicemen’s Testicular Tumor Environmental and Endocrine Determinants (STEED) for DDT exposure. Subjects in the highest serum *p,p*′-DDE quartile (>0.390 μg/g lipid) compared to those in the first serum *p,p*′-DDE quartile (0.157 μg/g lipid) supported increased risk of TGCT in relation to exposure to DDE and PCBs (78). On the other hand, DDE was not associated with TGCT in a case–control study of 876 adult men in Washington State, U.S. (79). Finally, several small studies have suggested an association between PCB exposure and prostate cancer (80, 81), whereas no association was reported between PCBs and prostate cancer in a recent Canadian study of 79 cases and 329 age frequency matched controls (82).

As far as genotoxic and mutagenic effects of BPA are concerned, most studies are carried out in *in vitro* systems, which do not mimic the *in vivo* environment. BPA has been evaluated in standard screens for mutagenicity including the Ames test, mouse lymphoma, sister chromatid exchange, and mammalian gene mutation assay. Most of the results indicated that BPA is not mutagenic (83). However, some reports have indicated that BPA has the potential to induce point mutation, double stranded DNA breaks, and aneuploidy (57, 58). The National Toxicology Program (NTP) has evaluated the carcinogenic activity of BPA and concluded that it was not a robust carcinogen in the context of adult exposure (84). However, careful analysis of the same data documented several shortcoming of the NTP study with respect to effects observed on hematology of mice and testicular tumors, age of animal as well as use of strain of rats and mice and their susceptibility to carcinogenic agents (85). Recent studies have shown that prenatal exposure to BPA causes hyperplasia of prostate in male rats resulting in greater risk of prostate cancer (86). Currently, there are few *in vivo* genotoxicity studies carried out in bone marrow cells of mice upon BPA exposure at different time intervals (1–5 days), which document that BPA failed to induce chromosomal aberrations and micronuclei formation (87, 88). Based on a few *in vivo* studies, it is impossible to draw a definite conclusion about genotoxic activity of BPA as it is estrogenic in nature.

Most evidence depicting cancer risk associated with exposure to EDC is limited to cellular and animal models. Although some evidence shows associated TGCTs, there is no conclusive evidence to indicate an increased risk of testicular cancer in men exposed to EDCs.

## Mechanism(s) of Action on EDCs Affecting the Male Reproductive System

Endocrine disrupting chemicals have long been known for their estrogenic properties and the ability to compete with endogenous steroid hormones binding to receptors. EDCs were found to disturb human male steroidogenesis, which alters reproductive hormones, a critical factor in spermatogenesis. Recent studies provide new insights about other mechanisms, such as oxidative stress, genetic susceptibility, and epigenetic effects, related to EDCs’ involvement with detrimental reproductive health outcomes.

### Steroidogenesis

Endocrine disrupting chemicals can act as anti-androgens, anti-estrogens, and steroidogenic enzyme inhibitors that interfere with steroid action/production as the mechanism to alter male reproductive health. Also, EDCs can interact with thyroid hormones and their receptors or with the brain and the hypothalamo-pituitary axis (89). Certain EDCs could inhibit the enzymes involved in steroidogenesis, which leads to the reduction of hormones. Urinary phthalate metabolites and BPA levels were negatively associated with testosterone levels (29, 90), follicle-stimulating hormone (FSH) (91), and luteinizing hormone (LH) (50). Phthalate esters were observed to exert a direct effect on Leydig cell or Sertoli cell structure with correlation of the *in vitro* and *in vivo* systems (92). DEHP was also observed to altered Sertoli and Leydig cell function during development and inhibit testosterone production (93). DEHPs can exert their anti-androgenic action by directly inhibiting testosterone synthesis in Leydig cells (94), which has been proposed to be a result of cytochrome CYP 17 dysfunction (95). Some phthalates have also been shown to disrupt the patterns of gene expression that regulate cholesterol and lipid homeostasis or insulin signaling, which could also result in lower testosterone synthesis (96). In addition, certain phthalate monoesters may interfere with the ability of Sertoli cells to respond to their normal endogenous ligand of FSH (97). The action site of the phthalate monoester was at the coupling of the FSH receptor-ligand complex to the transducing G-protein within the Sertoli cell membrane (97). Among phthalate metabolites, MEHP did not affect steroidogenesis in the H295R steroidogenesis assay nor alter testosterone production in MA-10 cells (98, 99).

Bisphenol A was also observed to have an inverse relationship with reproductive hormones. A recent study showed that the reduction of testosterone could be due to BPA-induced inhibitory effect on the activity of ATP-binding cassette transporters of the cellular membrane of testicular tissues (100). BPA has affinity to bind to estrogen receptors [estrogen receptor alpha (ERα) and estrogen receptor beta (ERβ)]. Knockout models for estrogen receptors have shown that they are the pivotal players required for spermatogenesis, suggesting that estrogen plays an important role in testicular development and spermatogenesis (101).

Dichlorodiphenyltrichloroethane could cause a decrease in transport of testicular androgen as a result of enhanced degradation (102). DDT and its metabolites (*o,p*′-DDT, and *p,p*′-DDE) can inhibit endogenous ligand binding to the estrogen and androgen receptors. PCBs can disrupt estrogen receptor function by mimicking the natural ligand and acting as an agonist (103).

### Oxidative stress

Recent evidence suggests oxidative stress may be one of the mechanisms associated with the effect of phthalates acting as an anti-androgenic compound on male reproductive health. Phthalates, mainly DEHP, DBP, or DEP, have been reported to alter the activities of marker testicular enzymes of laboratory animals associated with the specific events of spermatogenesis, inducing ROS production, lipid peroxidation, and apoptosis of spermatocytes. One possible mechanism is that ROS generation might correlate with DEHP-induced Ca^2+^ entry, potentially through the Ca^2+^-mediated activation of the nicotinamide adenine dinucleotide phosphate (NADPH) complex (104). Recently, limited epidemiological studies have also reported that phthalate metabolites are associated with oxidative stress. Fong et al. assessed the association between urinary phthalate metabolites in polyvinyl chloride workers. After adjustment for age, smoking status, and coffee consumption, sperm apoptosis and ROS generation were positively associated with urinary MEHHP, MEOHP, and MEHP (105). Specific signaling pathways mediate increased oxidative stress are needed to confirm oxidative stress as a mechanism for the effect on phthalate.

Bisphenol A could induce oxidative stress in fish spermatozoa *in vitro*, which results in accumulation of LO and CP, together with the modification of antioxidant system activity. These oxidative responses were associated with spermatozoa quality depression, as measured by a decrease in the values of spermatozoa motility and velocity (106). Adverse effects of the monomer in male rats may be due to induction of oxidative stress in sperm (107). With limited evidence to date, it is pre-mature to recognize oxidative stress as the mechanism associated with the effect of BPA on male reproductive capacity.

### Epigenetic mechanism

Possible negative actions on progeny via epigenetic toxic mechanisms have recently been suggested. Epigenetic changes include multiple mechanisms such as DNA methylation, histone modifications, and non-coding RNAs, which regulate gene expression without affecting the gene sequence (108). Epigenetic modifications via DNA methylation are permanent changes, which are transmitted to next generations (109). As indicated earlier, BPA has reduced affinity to bind to estrogen receptors that could lead to gene transcription and regulate DNA methylation in various disease states. Doshi et al. demonstrated that neonatal exposure of rats to BPA led to aberrant DNA methylation in testis, indicating methylation mediated epigenetic changes as one of the possible mechanisms of BPA-induced adverse effects on spermatogenesis and fertility (110). Wu et al. observed that DEHP-induced changes in DNA methylation, especially within CpG islands, and suggested that changes in DNA methylation may be one possible mechanism of DEHP-mediated testicular toxicity (111). Although emerging observations further expand the possibility of epigenetic as a toxic mechanism of EDCs but are yet to be verified in human studies.

## Future Research Needs

Current evidence has shown variability in study findings regarding the relationship between endocrine disruptors and male reproductive health. Some possibilities explain the variability. First of all, many of the potential exposure–response relationships described here have not been adequately explored. Second, there are differences among the studies, including differences in sample size, study design, study populations, life stage, data analysis approaches, and/or strategies for attaining data on exposure, endpoint, and important covariates. Third, limitations are inherent in epidemiological studies. For example, humans are not exposed exclusively to the chemical being investigated, but instead to a mixture of chemicals, some of them acting through common pathways. In addition, no single compound can act as a surrogate or marker for the others because the contaminant profile varies among individuals. Finally, different chemicals may have different toxicokinetics.

Current evidence has provided a better understanding of the impact of exposure to endocrine disruptors on male reproductive health along with possible mechanisms. However, future studies are needed to address inconclusive outcomes: (1) a well-defined epidemiology study with cohorts of men in various populations is required to evaluate the potential effect of external factors on male reproductive health. Such a study should not be limited to the analysis of sperm concentration, as this may not be the best biomarker of testis function and human fertility; (2) human exposure assessment data does not uniformly support toxicity of the substances at environmental concentrations. There is a need to develop methods to better study mixtures of endocrine disruptors, such as exposure to multiple phthalates at different levels and how they may act additively or synergistically, or even antagonistically. Statistical methods should incorporate the biological activity of the different phthalate metabolites, both the monoesters and oxidative metabolites; (3) there is a clear need for chemical and biochemical approaches aimed at a better understanding of the mechanism of action of xenoestrogens with regard to the low-dose effects revealed during developmental exposure. These approaches encompass several areas of study, such as signal transduction via membrane and nuclear ER, and analytical chemistry to measure these chemicals and their metabolites in tissues. Humans are exposed to a variety of endocrine disruptors acting through many different pathways at different times during their development. This poses two problems for consideration: interactions among chemicals acting through a common pathway and a single chemical affecting different pathways. A limited number of studies have shown that certain EDCs could induce epigenetic change. More studies are needed to confirm the results and any association with altered reproductive dysfunction and the etiology of congenital anomalies by identifying tissue-specific genes with changes in DNA methylation; and (4) identify susceptibility factors that may increase risk of adverse effects following exposure to EDCs. Genetic factors may modify the exposure–dose relationship by altering the metabolism or excretion of EDCs. Additionally, genetic factors may modify dose–response relationships by altering the biological response to a given internal dose. More research in this area of susceptibility is critical to our understanding of human health risks.

## Conflict of Interest Statement

The author declares that the research was conducted in the absence of any commercial or financial relationships that could be construed as a potential conflict of interest.
